# Immune status for monitoring and treatment of bladder cancer

**DOI:** 10.3389/fimmu.2022.963877

**Published:** 2022-09-08

**Authors:** Shen Pan, Shijie Li, Yunhong Zhan, Xiaonan Chen, Ming Sun, Xuefeng Liu, Bin Wu, Zhenhua Li, Bitian Liu

**Affiliations:** ^1^ Department of Radiology, Shengjing Hospital of China Medical University, Shenyang, China; ^2^ Department of Urology, Shengjing Hospital of China Medical University, Shenyang, China

**Keywords:** bladder cancer, BCG, immunotherapy, immune cells, type 2 immunity

## Abstract

The high recurrence rate of non-muscle invasive bladder cancer (BC) and poor prognosis of advanced BC are therapeutic challenges that need to be solved. Bacillus Calmette-Guerin (BCG) perfusion was the pioneer immunotherapy for early BC, and the discovery of immune checkpoint inhibitors has created a new chapter in the treatment of advanced BC. The benefit of immunotherapy is highly anticipated, but its effectiveness still needs to be improved. In this review, we collated and analysed the currently available information and explored the mechaisms by which the internal immune imbalance of BC leads to tumour progression. The relationship between immunity and progression and the prognosis of BC has been explored through tests using body fluids such as blood and urine. These analytical tests have attempted to identify specific immuyne cells and cytokines to predict treatment outcomes and recurrence. The diversity and proportion of immune and matrix cells in BC determine the heterogeneity and immune status of tumours. The role and classification of immune cells have also been redefined, e.g., CD4 cells having recognised cytotoxicity in BC. Type 2 immunity, including that mediated by M2 macrophages, Th2 cells, and interleukin (IL)-13, plays an important role in the recurrence and progression of BC. Pathological fibrosis, activated by type 2 immunity and cancer cells, enhances the rate of cancer progression and irreversibility. Elucidating the immune status of BC and clarifying the mechanisms of action of different cells in the tumour microenvironment is the research direction to be explored in the future.

## Introduction

There are an estimated 500,000 new cases of bladder cancer (BC) and 200,000 related deaths worldwide each year. In the US alone, > 80,000 new cases of BC and 17,000 deaths occur each year (1–3). In recent decades, the treatment of BC has improved gradually (4, 5) and the latest major development started in 1977 with the introduction of Bacillus Calmette-Guerin (BCG) in therapeutic management (6). In the past few years, tumour molecular profiling and immune checkpoint blockade have led to unprecedented progress in the treatment of BC (7–11). However, immune checkpoint inhibitors (ICIs) only provide survival benefits to 20–30% of BC patients (12–15). However, this limited effectiveness has not prevented ICIs from replacing previous chemotherapy regimens as the first and second-line treatment of BC (16–18). Immune-related therapies have featured prominently in the history of BC treatment. Researchers have focused more attention on human immune surveillance and elimination mechanisms, with the goal of eliminating BC cells in the body. However, the effectiveness of immunotherapy for BC still needs to be improved.

The function of the cellular components in the BC microenvironment is related to their molecular subtypes; precise tumour status markers need to be further explored. The accumulation of high-throughput data has provided a strategy to elucidate the mechanisms underlying cancer development (19–21). The most representative data hosted on The Cancer Genome Atlas (TCGA), comprising chromatin landscape, transcriptome, epigenetic alterations, and clinical data (22–25). Based on multi-omics data analysis, muscle invasive BC (MIBC) has been divided into six molecular subtypes (26, 27), including the stroma-rich and basal/squamous subtypes, which have lower tumour purity.

In addition, the luminal-infiltrated subtype shows high expression of epithelial-mesenchymal transition (EMT) markers and high resistance to cisplatin-based chemotherapy (26, 28). EMT markers, such as Twist, Snail and Zeb1, also induce chemoresistance in cancer (29). In various malignant tumours, including urothelial carcinoma (UC), there is a positive correlation between T cell infiltration and expression of EMT-related genes (30). T cell infiltration has been revealed to not be directly involved in the effect of ICIs (31). Furthermore, the infiltration of immune and stromal cells, which decreases tumour purity, is related to BC progression and poor prognosis (32).

The immune status of BC is not only associated with its staging and progression but also closely related to the outcome of BCG treatment and chemotherapy. Changes in the immune status of the microenvironment of BC affect the recruitment of a variety of cells and cause fluctuations in immune cells in the blood and urine. Type 2 immunity, which mediates wound healing fibrosis, is related to the progression, recurrence, and refractory nature of BC. In this review, we have collated and analysed the above information and explored the reasons for tumour progression caused by the internal immune imbalance in BC.

## Miscellaneous cells similar to those involved in wound repair appear in the BC tissue

The urothelium, submucosa, and muscle layers are part of the bladder wall, bladder tumours mostly originate in the urothelium layer (33, 34). The innate immune system shows anti-tumour activity based on the detection of tumour-associated antigens (TAAs) and damage-related molecular patterns (DAMPs). TAAs and DAMPs are presented by antigen-presenting cells (APCs), such as dendritic cells (DCs) and macrophages, to cytotoxic lymphocytes, causing activation and subsequent tumour infiltration (35–37). A tumour is an incurable wound that is closely associated with traumatic inflammation (38). Cancer cells likely gain survival advantages by reducing the expression of the MHCI-like molecules, HLA-A, -B, and -C to evade cytotoxic lymphocytes (39). Nectin-4 is a TAA that is expressed on the surface of 97% of the UC. Currently, combinatorial treatment with pembrolizumab and enfortumab vedotin—an antibody-drug conjugate targeting nectin-4—has been approved by the FDA as a first-line treatment for cisplatin-ineligible patients with locally advanced or metastatic UC (40). Identifying and amplifying TAA signals and fully mobilising immune cells to attack tumour cells may be a future solution for BC.

The development of tumours and trauma involves the same cell types, including platelets (41). In trauma, the fascia acts as an external reservoir for the formation of a temporary stroma for scarring and provides numerous fibroblasts for rapid sealing of large open wounds (42). The submucosa of the bladder is similar to the fascia in trauma and stores numerous fibroblasts. The inflammatory infiltration in early BC spreads to the submucosa, which permanently activates fibroblasts and increases their conversion into cancer-associated fibroblasts (CAFs).

CAFs are stimulated by surrounding cells or activated by signal crosstalk in the tumour microenvironment (TME), similar to wound repair, and are mainly derived from resident fibroblasts (43–47). The downregulation of major histocompatibility (MHC) class I antigen of cancer cells and upregulation of programmed cell death 1 ligand 1 (PDL1) in the TME are the mechanisms underlying the evasion of cytotoxic T cells by cancer cells (48, 49).

**Figure 1:**
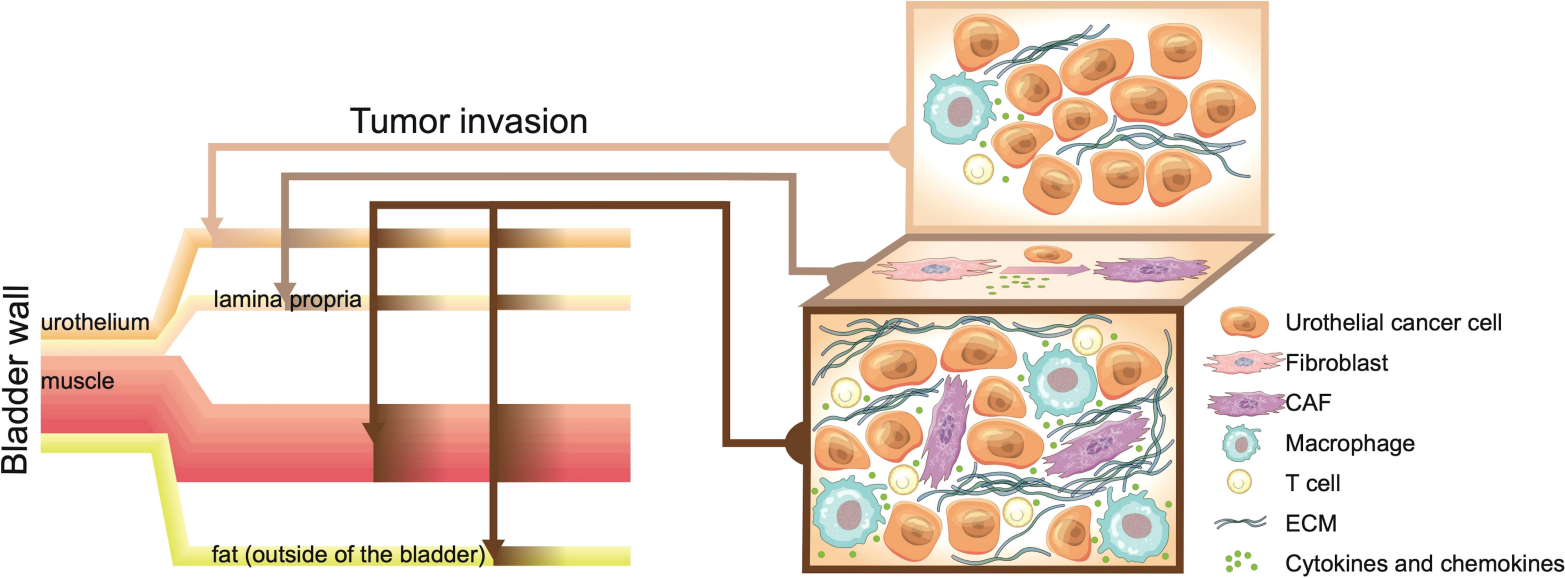
The bladder wall contains the urothelial layer, fibroblast-rich lamina propria, and muscle layer. Once bladder cancer (BC) tumours break through the bladder, they invade the external fat and adjacent tissues. Early BC is limited to the urothelial layer, with low immune cell infiltration and the highest tumour purity. Continued inflammatory infiltration and proliferation of cancer cells recruit and activate fibroblasts. The lamina propria, which is rich in fibroblasts, provides numerous cancer-associated fibroblasts (CAFs) for cancer progression and increases the heterogeneity of cancer tissues. The invasion or breakthrough of BC tumours through the lamina propria layer is similar to the repair process in the early stage of trauma, with infiltration of immune cells and fibroblasts. However, the immune infiltration and disordered stroma in BC continue to increase. Cancer cells that cannot be eliminated by the immune system exchange numerous signals with the complex cell environment, which makes the incurable “bladder scar” continue to recruit immune cells and fibroblasts.

The proliferation of tumour cells and their inability to be eliminated leads to long-term immune cell infiltration and further recruitment of fibroblasts in the stroma. This is completely different from established fibrosis where the monocyte-derived cell population is dismantled and degraded with the disappearance of inflammation after wound repair (50). In the progression of BC, the innate advantages of the different bladder wall layers and the infiltration of immune cells facilitate the recruitment of more stromal cells to enhance tumour heterogeneity and enrich the signal exchange in the TME (**Figure 1**).

## Overactive CAFs thrive in BC

CAFs are the main stromal cell type and are involved in the occurrence and development of various cancers (51). CAFs have multiple functions, including matrix deposition and remodelling, extensive interaction with cancer cells, and signal crosstalk with infiltrating immune cells (52, 53). In the immune-excluded phenotype, immune and tumour cells are separated by tumour stroma, preventing immune cells from killing tumour cells (54–56). Common markers of CAFs are vimentin, α-smooth muscle actin (SMA), fibroblast activation protein (FAP), S100 calcium-binding protein A4 (S100A4), and platelet-derived growth factor receptor-β (PDGFRβ) (57). The specific markers of CAFs in BC were identified using biocomputing; these included PDGFRβ (32).

Highly infiltrating CAFs and their markers are associated with the progression and poor prognosis of BC (58–61) and, along with high EMT/stromal-related gene expression, induce significant resistance to PD-1 blockade in UC (62). CAFs increased the resistance of BC cells to cisplatin through oestrogen receptor β (ERβ)/BCL2 signalling (63).

CAFs in BC can be divided into two subtypes, i.e., inflammatory and myo-CAFs (iCAFs and mCAFs, respectively) (64). iCAFs (PDGFRA+) strongly express various cytokines and chemokines, including CXCL12, IL-6, CXCL14, CXCL1, and CXCL2, which are very similar to the iCAFs described in pancreatic cancer (65). CXCL2—majorly from iCAFs—interacted with CXCR4 to induce tumour-associated macrophage (TAM) aggregation (66). Moreover, iCAFs—which are more closely related to the poor prognosis of BC than mCAFs (RGS5+)—might degrade and remodel the extracellular matrix, promote the proliferation of tumour and stromal cells, and recruit immune cells to tumours (64). However, mCAFs are considered the most abundant cell subtype in the stroma in many other types of tumours, which support the growth, survival, and metastasis of tumour cells (41, 67). Research on CAFs is still in its infancy, and the accurate classification of CAFs is crucial for the in-depth elucidation of their mechanism of action. Numerous experiments are still needed to verify this in the future.

## Cytotoxic immune cells

Immune surveillance mediates tumour clearance mainly through the action of cytotoxic CD8+ T cells (68–70). A high density of CD8+ T cell infiltration is associated with a good prognosis in BC (71, 72). However, T cell immunoreceptors with Ig and ITIM domain (TIGIT)-positive CD8+ T cells are related factors for the poor prognosis of MIBC and poor response to adjuvant chemotherapy (73). TIGIT, a new co-inhibitory receptor, downregulates the cytotoxicity and activation of T cells (74). Single-cell sequencing analysis has allowed for the comparison of CD8+ T cells in BC tumour tissue with normal tissues, the cell status and composition are not significantly different. In contrast, single-cell analysis of CD4+ T cells showed several tumour-specific states, including various states of Tregs, which also included clonally expanded cytotoxic CD4+ T cell (75). These CD4+ T cells kill autologous tumours in a major histocompatibility complex class II (MHCII)-dependent manner and are suppressed by Tregs (75). The previous concept defines the role of CD4+ T cells in anti-tumour immunity as indirect, mainly through Th cells to support CD8+ T cell-mediated tumour killing or Tregs to limit this class response (76, 77).

However, accumulating evidence has shown that some cytotoxic CD4+ T cells directly kill tumour cells and may play a key role in anti-tumour immunity (75). Mature CD4+ Th cells have also been shown to have phenotypic plasticity, inhibit CD8+ lineage genes under certain conditions, and exhibit MHCII-limited cytotoxicity (78). Because of its direct cytotoxicity, CD4+ T cell-mediated tumour killing has emerged as a unique mechanism of anti-tumour immunity.

In BC, two cytotoxic CD4+ T cell subpopulations have been identified, one expressing granzyme K (GZMK) and the other granzyme B (GZMB). CD4 GZMB subgroups express high levels of cytotoxic molecules GZMB, perforin, granulysin (Gnly), and natural killer (NK) cell granule protein 7 (NKG7), whereas CD4 GZMK expresses high levels of GZMK and lower levels of NKG7. Both the subsets produced high levels of the anti-tumour cytokine interferon (IFN)-γ and tumour necrosis factor (TNF)-α (75).

The unique biological role of cytotoxic CD4+ T cells still needs considerable investigation, especially the mechanism underlying cell population development and regulation and the mechanism by which cell populations contribute to tumour cell death. Uncovering the subpopulations of T cells and their mechanism of action would help improve the understanding of the response and drug resistance to ICIs, and the identification of novel targets for immunotherapy.

## Immunosuppressive cells

The role of immunosuppressive cells such as Tregs and macrophages in BC has not been fully elucidated. Tregs inhibit CD8+ T cell function by releasing immunosuppressive cytokines (including IL-10 and IL-35) (79, 80). Tregs in tissues play an important role in promoting tissue homeostasis and regeneration. Infiltration of FOXP3+ Tregs, which are involved in regression of inflammation, is associated with a better prognosis in BC (81, 82). However, high levels of C-C motif chemokine receptor 8 (CCR8)+ Tregs are associated with immunotolerance, low survival rates, and low chemotherapy response rates in MIBC (83).

CCR8, which mediates the immunosuppressive function, is an important chemokine receptor expressed in Tregs (84, 85). In BC, CCR8 maintains the stability of Tregs and enhances their inhibitory function by upregulating the expression of transcription factors FOXO1 and c-MAF (83). Blocking CCR8 decreases the stability of Tregs and enhances the therapeutic effect of PD-1 inhibitors (83). Human basic leucine zipper ATF-like transcription factor (BATF)+ CCR8+ Tregs from normal skin and adipose tissue have the same characteristics as non-lymphoid T follicular helper-like (Tfh-like) cells, with tissue regeneration and wound healing properties.

BATF+ CCR8+ Tregs also have the same characteristics as tumour-resident Tregs (86). BATF+ CCR8+ Tregs destroy the caspase recruitment domain family member 11- B-cell lymphoma 10- MALT1 paracaspase (CBM) signal body complex to modulate the function of Tregs and promote their production of IFN-γ. This not only inhibits tumour growth but also prepares the TME for successful immune checkpoint therapy (87). However, the exact role of Tregs in BC has not yet been clarified. Highly plastic macrophages, which usually exhibit an immunomodulatory M2 phenotype in tumours, are called TAMs.

Macrophages are polarised to the M2 phenotype and inhibit CD8+ T cell function after being manipulated by tumour-derived signals, including angiopoietin-2, macrophage-colony-stimulating factor (M-CSF), CCL2, and vascular endothelial growth factor (VEGF) (88, 89). Although the role of TAM in BC has not been confirmed, its infiltration may affect angiogenesis, tumour grade, and prognosis (90, 91). The TAM subgroups of BC that express dendritic cell-specific C-type lectin (DC-SIGN) significantly activate multiple M2-like signalling pathways, which are associated with poor prognosis and chemotherapy resistance. Moreover, blocking DC-SIGN has been shown to reduce the secretion of inflammatory cytokines in TAM and enhance the cytotoxicity of CD8+ T cells to MIBC cells mediated by PD-1 inhibition (92).

## Immune escape or suppression: Antigen presentation, recognition, and monitoring

To elicit an effective anti-tumour response, antigen presentation must be successful in the following two aspects. First, cancer neoantigens must be absorbed by specific APCs, mainly DCs, and cross-presented to activate naive CD8+ T cells (93). Second, neoantigens must be directly presented by tumour cells to enable triggered CD8+ T cells to recognise and kill them (94). Tumours have developed a variety of mechanisms to reduce these steps of antigen presentation and evade immune recognition by inhibiting DC function and downregulating tumour cell MHC expression.

DCs are a group of immune cells that play a central role in antigen presentation and effective anti-tumour T cell responses. UC has been found to induce high expression of the inhibitory receptors (IR, B, and T lymphocyte associated) BTLA and T cell immunoglobulin and mucin-domain containing-3 (TIM-3) in DCs in blood and tumours and mediates the decline and dysfunction of cytokine secretion in DCs (95). The MHCI and MHCII antigen presentation pathways play an important role in controlling the immune response (96).

Cytotoxic immune cells in BC, namely CD4+ T and CD8+ T cells, killed tumour cells by recognising MHCII and MHCI, respectively (75, 97). Single-cell-sequencing results showed that the downregulation of MHCII in BC cells contributes to the formation of an immunosuppressive TME (64). The downregulation or absence of MHCI in the TME leads to the dysfunction of CD8+ T cells (95). Although complete downregulation of MHC presentation appears to be an attractive escape mechanism for tumours to evade immune recognition, the immune system has an important checkpoint to monitor the loss of MHC presentation.

NK cells detect the loss of MHC surface expression as a stress signal and target stressed cells (94). Therefore, tumours have also evolved more subtle immune evasion strategies to deplete NK cells without eliminating the surface expression of MHC. Increasing the concentration of IL-21 in the TME could reverse the failure of NK cells by activating the phosphoinositide 3-kinase (PI3K)-AKT-FoxO1 and signal transducer and activator of transcription 1 (STAT1) signalling pathways to improve the prognosis of advanced tumours (98).

## Liquid biopsy and BC

Liquid biopsy is an easily accessible, non-invasive technique that provides real-time information about cancer (99). Blood-perfusing cancer tumours are key carriers of related substances that can be used to detect the presence and progression of cancer (100–104). In BC, in addition to blood, urine also directly makes contact with the tumour and exfoliates tumour cells, which can be used in the diagnosis of UC (105). Extracellular vesicles (EVs) and cell-free DNA (cfDNA) in the blood and urine can be used to monitor the progression, relapse, and treatment response of BC (106–108).

EVs including exosomes serve as a medium for signal communication between cells in cancer tissues (109–113). Both EVs and cfDNA penetrate adjacent body fluid compartments (109, 114) and abnormal changes in their levels in liquid biopsy are related to the heterogeneity of cells in cancer tissues. In peripheral blood, the percentage of CD3+CD25+ T lymphocytes in BC patients is significantly higher than that in healthy controls (115). However, the phenotypic overlap between peripheral blood mononuclear cells (PBMCs) and tumour-infiltrating lymphocytes (TILs) is the lowest.

**Figure 2:**
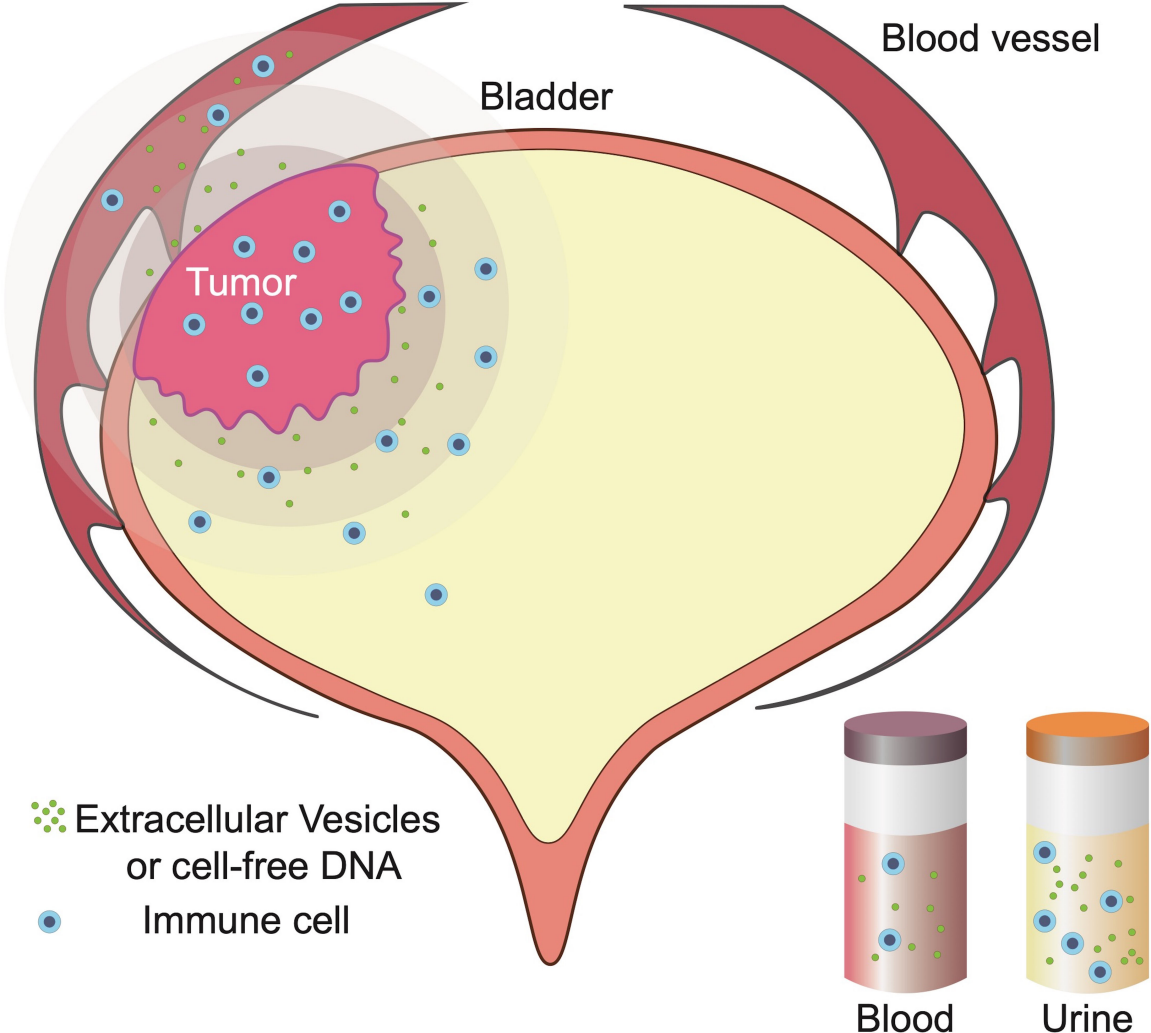
Blood flowing through bladder cancer (BC) tissue and urine that comes in contact with BC tissue reflects changes to the tumour microenvironment (TME). Currently, free cells and molecules in the urine may be shed from the tumour, similar to infused lymphocytes and molecules in BC.

The T cell checkpoint and T cell receptor (TCR) composition is similar between urine-derived lymphocytes (UDLs) and TILs, suggesting that UDLs are derived from the bladder tumour tissue. Viable CD3+ T lymphocytes, including CD8+, CD4+ FoxP3- (CD4eff), and CD4+ FOXP3+ (T regulatory cells, Tregs), have been detected in the urine of MIBC patients (116). However, the mechanism by which lymphocytes enter the urine is still unclear. UDLs can be used to accurately determine the immune status and lymphocyte composition in the TME of BC patients (**Figure 2**). In other words, the immune content in UDLs is closer to that in the tumour, while the immune content in blood does not decrease, but is greatly different from that in the tumour. This phenomenon remains to be clarified.

## Immunotherapy and BC

The mechanisms of action of BCG in non-muscle invasive bladder cancer (NMIBC) have not been completely elucidated (36). The intense inflammation induced by BCG involves a variety of chemokines and cytokines, which lead to infiltration of innate and adaptive immune cells, mainly neutrophils, T cells, and monocytes (117, 118). BCG Connaught and Tice are the two most widely used BCG strains in North America and Europe (119); different strains induce different immune responses.

Patients treated with BCG Connaught have significantly better recurrence-free survival rates than those treated with BCG Tice, which may be because its induction of T-helper 1 (Th1) and initiation of CD8+ T cells is superior to that of BCG Tice. The difference in treatment results may be attributable to the single nucleotide polymorphism (SNP) of the BCG strain (120). In addition, relapse after BCG treatment is associated with a lower ratio of T cells to monocytic myeloid-derived suppressor cells (M-MDSCs), which are known to inhibit immune function in tumours (121, 122).

M-MDSCs and the innate counterpart of Th2 cells, group 2 innate lymphocytes (ILC2), are detectable in urine after BCG treatment and may be recruited and induced by ILC2 to secrete IL-13. The ILC2/IL-13 axis mediates a mechanism that drives the immunosuppressive microenvironment, which is closely involved in the failure of BCG immunotherapy (123). Previous studies reported that the imbalance of Th1/Th2 cytokine IL-2/IL-10, that is, overproduction of IL-10, may contribute to the poor prognosis of NMIBC.

Controlling and reversing the production of IL-10 and promoting the overproduction of IL-2 would help eliminate tumours (124). In NMIBC patients who failed to respond to BCG treatment, the immunosuppressive cell subsets of traditional Tregs have a higher level of infiltration than other cells (125, 126). There is a significant difference in the expression of PD-L1 in Tregs expressing Forkhead box A1 (FoxA1) (127, 128).

The enrichment of PD-L1-positive Tregs that may be induced by IFN-β during BCG treatment increases, leading to a decrease in the therapeutic effect of BCG (127, 128). Compared with the expression of PD-L1 on tumour cells, T cells may be a more important non-classical source of PD-L1. Blocking the PD-1/PD-L1 interaction may increase immune-related BCG activity (128–130). Overall, the effect of BCG treatment mainly depends on the immune status of the local microenvironment and the detection of cytokines and immune cells in urine could predict the outcome of BCG treatment.

Because of the higher mutation spectrum, MIBC has a better response rate to ICIs and lower mutation rate than melanoma and non-small cell lung cancer (131–137). ICIs, such as PD-1 and PD-L1 inhibitors, have also become first-line treatment strategies for patients with advanced urothelial cancer who are not suitable candidates for platinum chemotherapy (57, 138). Nevertheless, the response rate to ICIs still needs to be improved and the sum of the objective and complete response rates in urothelial cancer is < 30% (3, 139, 140). The mechanism by which the immune checkpoint is blocked in BC remains to be further elucidated.

PD-L1 is not only expressed in cancer cells but also on the surface of a variety of immune cells, especially in lysosomal associated membrane protein 3 (LAMP3)+ DCs. The DC subgroup may directly inhibit CD8+ T cells (64). The high expression of PD-L1 in BC patients is favourable for treatment with PD-L1 inhibitors (8, 9, 141, 142). However, the type 2 immunity associated with tumour progression is also closely related to the expression of PD-L1. Group 2 innate lymphoid cells (ILC2s) expressing PD-L1 stimulate Th2 cells to increase GATA binding protein 3 (GATA3) expression and IL-13 production. As important participants in type 2 immunity, stromal cells were significantly associated with a lower response rate to PD-1 inhibitors (62).

The loss of PD-L1 on ILC2s can impair early Th2 polarisation and cytokine production (143). Surprisingly, the interaction between PD-L1 on ILC2s and PD-1 on CD4+ T cells did not inhibit the T cell response (143). However, we can confirm that type 2 immunity is detrimental to immunotherapy, including BCG and ICIs. The mechanism of action of immune checkpoints needs to be further elucidated, especially the markers of immune checkpoints on different cells in the immune microenvironment. In addition, the effect of the interaction among immune checkpoints on various functions between cells needs to be further investigated.

## Chemotherapy and BC

Although chemotherapy is still the first-line treatment for BC, its effects are limited (17). Three or four cycles of neoadjuvant chemotherapy (NAC) have been established as the standard adjuvant treatment for eligible MIBC patients (17, 144, 145). A meta-analysis of 11 clinical trials with a total of 3,000 patients showed the benefits of NAC with respect to improving the 5-year overall survival (OS) and reducing the risk of death (146). However, the correlation between the therapeutic effect of chemotherapy and the TME of BC patients has not been clarified. The gene expression profile was used to evaluate the role of the immune-related gene expression of BC patients in chemotherapy activity, and it was found not to be related to the effect of chemotherapy (147).

It may be that the expression of immune-related genes does not reflect the proportion of immune cell subsets, and therefore, the association between chemotherapy and immune status could not be identified. Chemotherapy can affect the internal TME in BC patients. For patients with urothelial cancer who have not received chemotherapy, the prognosis of failure of ICI therapy is often worse than for patients who received chemotherapy (13). FoxA1, which is a specific expression factor of Tregs induced by IFN-β, is related to the chemotherapy resistance of BC (127, 148).

Chemotherapy can enhance anti-tumour immunity, including stimulation of CD4+ effector T (Teff) cells and CD8+ Teff cells (149). NAC treatment decreases the expression of the depletion marker PD-1 in CD8+ and CD4+ Teff cells and increases that of the activation markers T-bet and the cytotoxic molecules granzyme B and perforin in the sentinel lymph nodes of BC patients (150). However, the phenotype and function of T cells in the sentinel lymph node in patients whose BC is not downgraded did not show these positive immune effects (150).

Long-term cancer control and complete remission may involve CD8+ T cell immune responses. The C-X-C motif chemokine receptor 3 alt (CXCR3alt)-CXCL11 chemokine system, which has stimulatory effects on CD8+ T cells, can predict the responsiveness of NAC in MIBC (151). CAFs, which play a role in the development of drug resistance in a variety of tumours, increase the resistance of BC cells to cisplatin by enhancing the transmission of ER β/Bcl-2 signals (63, 152). Therefore, the success of a refined NAC regimen to improve immunogenicity—perhaps by enhancing cytotoxicity or selecting low stroma infiltration—could be achieved by maximising the effectiveness of chemotherapy and immune promotion.

## Inflammation and BC

Inflammation has been identified as a driving factor for many cancers (153). Inflammation markers such as C-reactive protein (CRP) and IL-6 promote the progression of MIBC (154–156). Both CRP and IL-6 increase during the acute inflammatory phase and their levels reflect their severity (157–159). Active CRP increases the secretion of cytokine IL-6 (160). IL-6 is a cytokine with various physiological functions, including regulation of immune cell proliferation and differentiation (161). IL-6 not only amplifies cancer-causing chronic inflammation but also mediates the internal mechanism of tumour cells that drive cancer progression (162). IL-6 induces PI3K-AKT, mitogen-activated protein kinase (MAPK)/extracellular signal-regulated kinase (ERK), nuclear factor (NF)-κB, and STAT3 signalling (162).

CRP also has prognostic significance with respect to NMIBC. However, relapses confined to the bladder mucosa layer have a less effect on systemic inflammation and CRP is not ideal for predicting disease recurrence (163). Higher CRP levels are associated not only with poor prognosis for radical cystectomy, but also with poor prognosis after chemotherapy and radiotherapy in metastatic BC (164–168). However, CRP has been shown to have a tumour-killing activity in both *in vivo* and *in vitro* (160). Such results revealed the complexity of the role of CRP in BC. In tumours, CRP is involved in the recruitment of monocytes/macrophages, a phenomenon that increases the complexity of the TME (169–171). Perhaps CRP does not directly kill camouflaged cancer cells, as it is non-toxic to normal cells (160).

A gradual increase in inflammatory infiltration increases the number and various types of immune cells that accumulate in the TME. The progression of tumours is similar to wound healing. Following the accumulation of a considerable amount of inflammatory factors, the inflammatory phase subsides and the tissue repair process commences (172). Similar to the inflammation subsidence and tissue reconstruction phase, which enhance wound healing, the complex and multi-source signals present in the TME promote cancer progression.

## Type 2 immunity and BC

Typically, Th1 induces M1 macrophages and cytotoxic T cells to kill tumour cells in the pro-inflammatory type 1 immune response (173). Type 2 immunity is usually involved in the development of tolerance in the tumour environment, where Th2/M2-related cells are involved in immunosuppression and tumour progression (174, 175).

### M2 macrophages

M2 macrophages, often referred to as immunosuppressive cells, are also called TAMs (176). The M2 phenotype has been found to promote the progression of BC by suppressing inflammation (177). Squamous cell carcinoma-like BC, which dominates in MIBC, has greater M2 macrophage infiltration (178). Studies revealing the molecular mechanisms between BC and M2 macrophages are limited. Bone morphogenetic protein 4 (BMP4) secreted by BC cells induces M2 macrophage polarisation (179). Moreover, lysine-specific demethylase 6A (KDM6A), which is frequently mutated in BC, leads to activation of cytokine and chemokine pathways, which promotes the polarisation of M2 macrophages (180).

### Th2

Th2 is a predominant class of CD4+ Th cells characterised by the production of IL-4/IL-13 cytokines. There are few high-quality studies related to Th2 in BC. However, it is known that M2 macrophages recruit Th2 cells through the actions of CCL11/CCL22 (181–184). Th2 differentiation could be induced by IL-4 secreted by B cells, NK cells, naive CD4+ T cells, and mast cells (185). Furthermore, IL-4/IL-13 has been shown to contribute to cancer growth and metastasis (186, 187).

### IL-13

IL-13, which plays an important role in type 2 immunity, was found to be abnormally elevated in the urine of BC patients (188, 189). IL-13, described as an inhibiting inflammatory cytokine (190, 191), participates in immune surveillance in cancer, inhibits cancer cell apoptosis, and promotes tumour growth (192–194).

### CAFs

After BC tumours break through the muscle layer of the bladder or metastasise to distant sites, the CAFs and extracellular matrix (ECM) in the TME increases significantly (32, 64). However, excessive deposition of fibroblasts and ECM during wound healing can lead to scarring or fibrosis (195–198). The study of the wound healing process has considerably facilitated the understanding of cancer progression. Excessive type 2 immune activation involves Th2 cells and IL−4- and IL−13−activated M2 macrophages, which recruit more fibroblasts and cause excessive ECM deposition by IL-4/IL-13 and fibroblast growth factor (FGF)/PDGF, respectively. The progression of BC reduces the purity of the tumour and increases the proportion of stroma in the tumour (26, 199). The proliferation rate of CAFs, the main component of the stroma, has been shown to be faster in BC than under normal conditions, which contributes greatly to the growth and increased heterogeneity of the tumour.

**Figure 3:**
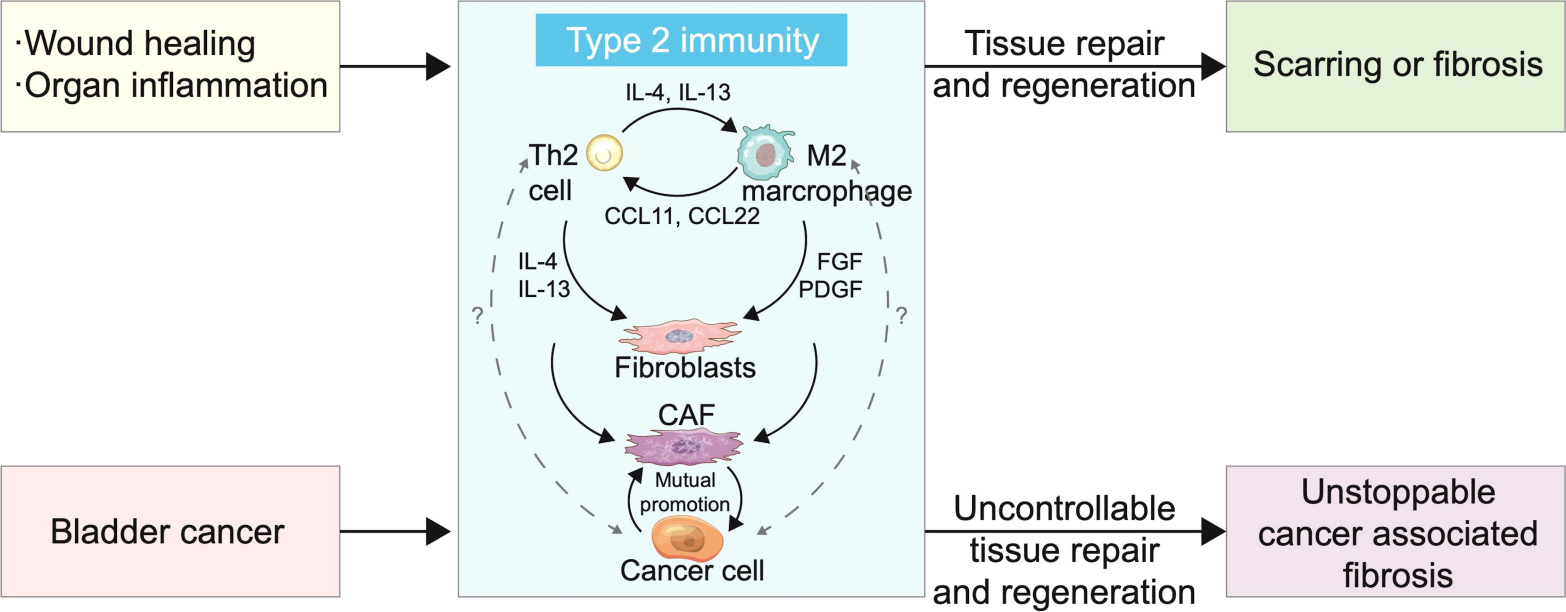
Type 2 immunity and bladder cancer (BC) cells cause rapid and persistent progression of cancer-associated fibrosis. Scarring or fibrosis caused by wound healing or inflammation of organs is associated with overactivation of type 2 immunity. T helper 2 (Th2) cells and M2 polarized macrophages promote mutual differentiation and jointly recruit fibroblasts. The proportion of M2 cells and cancer-associated fibroblasts (CAFs) in BC increased with tumour progression and interleukin (IL)-13 level increased significantly in tumours, which suggests that BC was closely related to type 2 immunity. CAFs have shown signal crosstalk with cancer cells in a variety of cancers, promoting differentiation, proliferation, and metastasis. Cancer cell involvement makes type 2 immunity persistent and uncontrollable, resulting in tumour metastasis.

In addition to the activation of type 2 immunity, the proliferation of CAFs is also related to the signals provided by cancer cells in the TME. BC cells trigger the differentiation of fibroblasts into CAFs through exosome-mediated transforming growth factor (TGF)-β transport and activation of the SMAD pathway (200). In oral squamous cell carcinoma, gastric cancer, and liver cancer, CAFs promote the proliferation and metastasis of cancer cells through exosomes (201–203). The signals produced by cancer cells disrupt the immune microenvironment and convert the type 2 immune response, which is targeted at repairing and maintaining homeostasis, into a persistent source of malignant tumours (**Figure 3**).

## Next generation therapeutics

The use of ICIs has impacted the treatment of BC. Antibody-drug conjugates (ADCs), such as enfortumab vedotin, are approved for patients locally advanced or metastatic UC who were unresponsive to PD-1/PD-L1 therapy (204). ADCs—agents targeting Trop-2, HER2, and EpCAM—are undergoing clinical evaluation for UC (205) and have the potential to emerge as next generation UC treatments. Similarly, some bispecific antibodies and fusion proteins in the research and development phase, such as T cell engagers, Treg depleting ATOR-1015, and super agonistic cytokine traps ALT-803, are likely to introduce a change in the manner UC is treated (206–210). The next generation of treatments, based on activation of the immune system and recruitment of effector cells to kill cancer cells while minimising the side effects, and a variety of other treatments, are undergoing clinical trials for BC (211).

## Conclusions and future directions of BC research

Immune status is the main factor determining prognosis in BC and is evaluated using relatively invasive body fluid testing (99, 106). The significance of fluctuations in immune cells and cytokines in blood and urine in patients with BC has not been fully demonstrated. In the future, it may be possible to predict the prognosis and guide treatment of BC patients by analysing the immune state of bodily fluids, which are reflective. The role of type 2 immunity and the related cytokines in the host immune system and inflammatory diseases is to suppress type 1 and Th17-driven inflammation and participate in the repair and regeneration of damaged tissue (181, 182, 212).

Overreaction of type 2 immunity leads to pathological fibrosis, such as liver and pulmonary fibrosis (213–216). The mechanism underlying the development of fibrotic disorders mediated by type 2 immunity is unclear, but signals that continuously activate tissue repair are known to contribute to this condition. The involvement of cancer cells further complicates the imbalance of type 2 immunity in the TME. Studies have shown that blocking type 2 immune cytokines, such as IL-4 and IL-13, can interfere with the inhibition of type 1 and Th17-driven inflammation, which can induce considerable neutrophilic inflammation.

**Figure 4:**
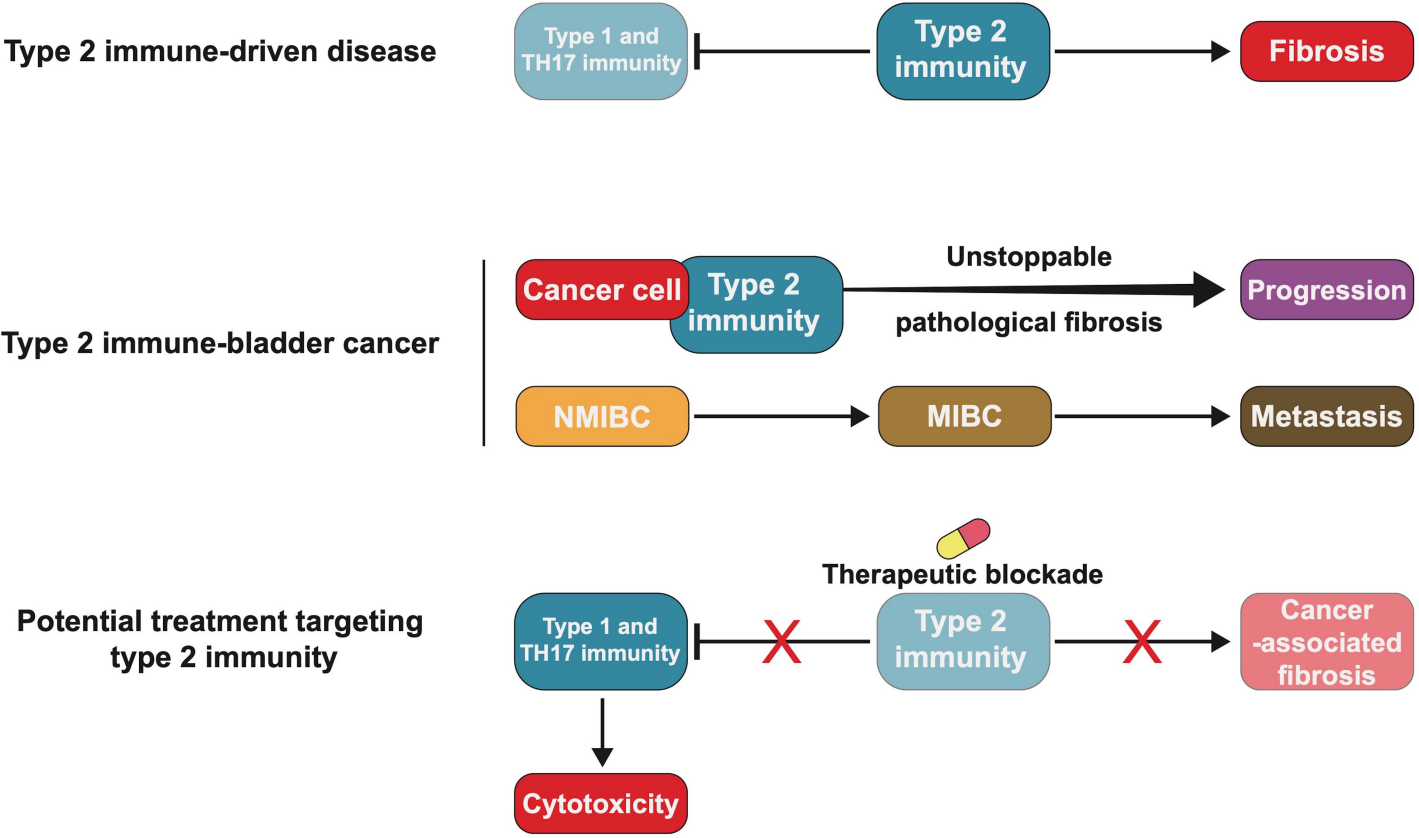
Blocking type 2 immunity may be an effective treatment for bladder cancer (BC). Type 2 immune-driven diseases, such as scarring and organ fibrosis, are caused by excessive recruitment of fibroblasts and inhibition of the cytotoxicity of type 1 and T helper 17 (TH17) immunity. BC cells interact with type 2 immunity-related molecules, making pathological fibrosis persistent and causing tumour progression or metastasis. Blocking type 2 immunity could prevent pathological fibrosis and in BC may be worth future exploration and study.

In the experimental myeloid schistosomiasis and pulmonary cell tumour model, blocking IL-13 significantly reduced fibrosis, but type 1 and Th17-driven inflammation simultaneously exacerbated liver and lung damage (217). Cancer-related fibrosis of the TME, likely induced by the blockade of type 2 cytokines, slows down the pathological fibrosis associated with the uncontrollable nature of tumour and active cytotoxicity of type 1 immunity (**Figure 4**). However, there may also be excessive accumulation of type 1 and Th17-related inflammatory molecules, with considerable inflammatory fluids providing nutrition for BC cells, which promotes their proliferation and metastasis.

In the allergenic mouse model, the dual blockade of IL-13 and IL-17A protected mice from acidophilic and neutrophilic inflammation and the damage caused by rebound inflammation (217, 218). This also prompted us to attempt to treat BC using dual blockade (217, 219). The study of the immune status and mechanism of action of related molecules in BC is still in its infancy and identification of the immune stage and control of immune status should be the primary focus of future research. The correlations among multiple immune, matrix, and cancer cells need to be studied and elucidated in future studies.

## Author contributions

BL, SP, and SL conceptualised and designed the study and wrote the article. ZL, XL, and BW collected the documents and reviewed the manuscript. YZ, MS and XC revised the manuscript. All authors contributed to the article and approved the submitted version.

## Funding

The present study was supported by 345 Talent Project from Shengjing Hospital of China Medical University (Grant Nos. M0312 and M0340), and the Doctoral Scientific Research Foundation of Liaoning Province (Grant No. 2020-BS-120).

## Conflict of interest

The authors declare that the research was conducted in the absence of any commercial or financial relationships that could be construed as a potential conflict of interest.

## Publisher’s note

All claims expressed in this article are solely those of the authors and do not necessarily represent those of their affiliated organizations, or those of the publisher, the editors and the reviewers. Any product that may be evaluated in this article, or claim that may be made by its manufacturer, is not guaranteed or endorsed by the publisher.

## Glossary

**Table:**

BC	Bladder cancer
BCG	Bacillus Calmette-Guerin
IL	Interleukin
ICIs	Immune checkpoint inhibitors
TCGA	The Cancer Genome Atlas
MIBC	Muscle invasive bladder cancer
EMT	Epithelial-mesenchymal transition
TAA	Tumour-associated antigens
DAMPs	Damage-related molecular patterns
APC	Antigen-presenting cells
DCs	Dendritic cells
CAFs	Cancer-associated fibroblasts
TME	Tumour microenvironment
MHC	Major histocompatibility
PDL1	Programmed cell death 1 ligand 1
EVs	Extracellular vesicles
cfDNA	Cell-free DNA
PBMCs	Peripheral blood mononuclear cells
TILs	Tumour-infiltrating lymphocytes
TCR	T cell receptor
UDLs	Urine-derived lymphocytes
NMIBC	Non-muscle invasive bladder cancer
Th1	T-helper 1
SNP	Single nucleotide polymorphism
M-MDSCs	Monocytic myeloid-derived suppressor cells
ILC2	Group 2 innate lymphocytes
FoxA1	Forkhead box A1
IFN	Interferon
NAC	Neoadjuvant chemotherapy
OS	Overall survival
TIGIT	T cell immunoreceptor with Ig and ITIM domain
GZMK	Granzyme K
GZMB	Granzyme B
Gnly	Granulysin
NK	Natural killer
NKG7	Natural killer cell granule protein 7
TNF	Tumour necrosis factor
TIM-3	T-cell immunoglobulin and mucin-domain containing-3
PI3K	Phosphoinositide 3-kinase
STAT1	Signal transducer and activator of transcription 1
CCR8	C-C motif chemokine receptor 8
BATF	Basic leucine zipper ATF-like transcription factor
CBM	Caspase recruitment domain family member 11- B-cell lymphoma 10- MALT1 paracaspase
TAM	Tumour-associated macrophages
M-CSF	Macrophage-colony-stimulating factor
VEGF	Vascular endothelial growth factor
DC-SIGN	Dendritic cell-specific C-type lectin
SMA	α-smooth muscle actin
FAP	Fibroblast activation protein
S100A4	S100 calcium-binding protein A4
PDGFRβ	Platelet-derived growth factor receptor-β
ERβ	Oestrogen receptor β
CRP	C-reactive protein
MAPK	Mitogen-activated protein kinase
ERK	Extracellular signal-regulated kinase
NF	Nuclear factor
ECM	Extracellular matrix
FGF	Fibroblast growth factor
TGF	Transforming growth factor
LAMP3	Lysosomal associated membrane protein 3
GATA3	GATA binding protein 3
